# Religion, Spirituality, Well-Being and Praying the Rosary: Results of a Cross-Sectional Study from Germany

**DOI:** 10.1007/s10943-024-02210-5

**Published:** 2024-12-14

**Authors:** Michael Teut, Benno Brinkhaus, Barbara Stöckigt, Sylvia Binting, Michael K. Elies, Christian Zwingmann, Florian Jeserich

**Affiliations:** 1 Institute for Social Medicine, Epidemiology and Health Economics, Charité – Universitätsmedizin Berlin, Corporate Member of Freie Universität Berlin, Humboldt-Universität zu Berlin, and Berlin Institute of Health, Berlin, Germany; 2 Laubach, Germany; 3 The Protestant University of Applied Sciences Rhineland-Westphalia-Lippe, Bochum, Germany; 4 Contilia Academy, Wissollstrasse 11, Mülheim an der Ruhr, Germany

**Keywords:** Rosary, Prayer, Well-being, Meditation

## Abstract

Rosary prayer is a popular Catholic meditative prayer practice and has been used since the thirteenth century. The aim of this study is to describe characteristics, prayer practice, religiosity and piety of those practicing the prayer and to investigate whether and how religion/spirituality (R/S) and well-being are related in this specific population. An online cross-sectional survey was performed between June and September 2022 which included items on sociodemographic data, prayer practice, well-being, religiosity and spirituality, transpersonal trust and spiritual meaning. Results were described descriptively, and a possible relationship between R/S and well-being was examined using correlational and moderator analyses. In total, 164 participants who pray the Rosary completed the online questionnaire. A total of 92% of the participants belonged to the Roman Catholic Church, and 61% of the sample were women. A majority of participants reported to be very religious/spiritual (36%) or quite religious/spiritual (47%). Most participants experienced the prayer as a calming and relaxing practice, which slows down the breathing and the awareness in the prayer being focused on the prayer beads. Most of the participants expressed a high-to-very high transpersonal trust. All R/S measures used in this study are highly intercorrelated (*r* between 0.64 and 0.91) and show similar small-size associations (*r* between 0.10 and 0.20) with well-being. Moderator analysis shows that the connection between R/S and well-being was stronger among those with more positive prayer experiences. Subjectively perceived positive prayer experiences may act as an amplifier or emotional affirmation of the “rightness” or “effectiveness” of one’s faith and this very amplification may have a strengthening effect on the relationship between R/S and well-being. The interaction of R/S and well-being in Rosary praying and other meditative techniques should be a major topic of future research.

## Introduction

Rosary prayer is a Catholic meditative prayer practice that can be historically tracked back to the first written version by the Cistercian Abbot Stephen of Sallay in the thirteenth century. In the fifteenth century, the Carthusian monk Dominic of Prussia formed the “clausulae,” which summarized the life of Jesus in 50 sentences, which are still in use today. Later, a much briefer 15 sentence version was introduced by Alanus de Rupe, which made the Rosary prayer very popular with common people (Stöckigt et al., 2021; von Brockhusen, 1989).

The individual beads of the rosary are used to guide the prayer, bead by bead, through the life of Jesus and Mary by means of short prayer sentences (mysteries) and to pray and meditate on the individual significant stages of their lives.

Today, the mystery sentences are grouped in three sets, being arranged around the incarnation of God in Jesus (“Joyful Mysteries”), the passion of Jesus (“Sorrowful Mysteries”) and his resurrection (“Glorious Mysteries”) (Graber, 1976; Winston-Allen, 1997). The most popular Rosary prayer cord includes 59 beads and a cross, each bead represents the Lord’s prayer, Hail Mary Prayer or Glory be Prayer, in regular sequence including five mystery sentences and the three mysteries in sequence.

A popular form of the Rosary prayer begins with the drawing of the cross, followed by praying the Apostles' Creed, Our Father, Hail Mary, Glory Be. Then, the prayer of one of the following three mysteries begins: 1. Joyful Mysteries (The Annunciation, The Visitation, The Nativity of Our Lord Jesus Christ, The Presentation, Finding Jesus in the Temple), 2. Sorrowful Mysteries (The Agony in the Garden, The Scourging at the Pillar, Jesus is Crowned with Thorns, Jesus Carries His Cross, The Crucifixion) or 3. Glorious Mysteries (The Resurrection, The Ascension of Jesus to Heaven, The Descent of the Holy Spirit, The Assumption of Mary in Heaven, Mary is Crowned Queen of Heaven and Earth). The prayer closes with the prayers Our Father, Hail Mary and Glory Be and with drawing the cross. The mysteries, which each consist of 5 sets of prayers or stations, are often varied according to the days of the week. There are several additional mystery prayers and many variations of the practice across several catholic cultures and countries.

Generally, spirituality can be understood as a social determinant of health which is linked to human goods and is deeply valued by people and their communities. Spirituality includes a sense of ultimate meaning, purpose, transcendence and connectedness (Balboni et al., 2022; Long et al., 2024).

### Current State of Research

The empirical literature on the possible relationship between prayer and health has grown rapidly (e.g., Breslin & Lewis, 2008; Masters & Spielmans, 2007; Simão et al., 2016). Most studies report a positive correlation between prayer and various positive health outcomes (e.g., Jarego et al., 2023; Meisenhelder & Chandler, 2001) or inverse correlations between prayer and negative indicators of health (e.g., Roberts et al., 2009; Anderson & Nunnelley, 2016). In the entire research field, two research endeavors can be roughly distinguished. One line of research is concerned with developing cross-religious typologies of prayer and discussing the positive effects that can be attributed to prayer as a religious practice that is widespread across cultures (e.g., Poloma & Gallup, 1992; Upenieks, 2023; Whittington & Scher, 2010). In terms of the history of science, Friedrich Heiler (1918) laid the foundation for this branch of research with his comparative study “Das Gebet.” In this book, he defines prayer as the central phenomenon of religion and presents a classification of prayer across time, cultures and religions. Another line of research moves away from this comparative meta-perspective and places the cultural and religious specifics of the prayer practices of certain groups at the forefront of scientific analysis. Prominent examples are studies of the connection between health/well-being and the ritual practice of prayer (salat) in Islam (e.g., Doufesh et al., 2014; Saleem et al., 2021; Sarkingobir et al., 2022) or Buddhist mindfulness meditation, which has been scientifically tested for its health benefits since Jon Kabat-Zinn promoted Mindfulness-Based Stress Reduction (MBSR) as a clinical intervention (Kabat-Zinn, 2003) at the latest (e.g., Fisher et al., 2023; Geiger et al., 2023; Wang et al., 2024).

While the empirical study of the Rosary prayer has rarely taken place, the relationship between prayer in general and aspects of health/well-being in German samples has already been the subject of empirical work. In addition to statistical surveys that show, for example, that Germans tend to pray less in comparison to other European countries (Hank & Schaan, 2008), and the quantitative studies by Büssing et al. (2016, 2020, 2021) are particularly worth mentioning in our research context. For example, Büssing et al. (2016) show that it is not only the frequency of prayer that matters, but also the form of prayer (private or public) and the experience of the transcendent. As this study was conducted in a very specific group (Roman Catholic pastoral workers), the results are particularly instructive for the present study, in which almost exclusively Catholic Rosary prayers were surveyed.

Against this background, our research question arose as to whether and to what extent the ritual practice of praying the Rosary in Germany could have similar positive effects on the general well-being of people of Christian faith. To our knowledge, such an empirical study has not yet been conducted in Germany, which is why a qualitative pilot study was initially carried out (Stöckigt et al., 2021), on which the present quantitative study is based.

### Qualitative Pilot Study

In a recently published qualitative study, we investigated experiences and perceived effects of the Rosary prayer on health, well-being, spirituality and religiosity in interviews with ten Catholic adults who regularly prayed the Rosary (Stöckigt et al., 2021). In this study, our interview participants described experiencing a sense of inner stability and peace and an inner connection with the Divine and with Mary as a mediator before God as consequences of the prayer practice.

The Rosary prayer practice may be helpful as a coping strategy and also for developing a Christian way of life with a humble attitude and a devotion toward and trust in God and his will. The Rosary helps balancing submission to God's will with receiving emotional support through the structure of Rosary prayer. While accepting that one is not in control of illness or death, one can, with a sense of relief and without pressure, actively do something for one's health in form of praying (Stöckigt et al., 2021).

Most interview participants described to have become familiar with the prayer through family members during childhood, especially through female relatives and that the prayer helped them in coping with difficult life events and circumstances. Holy Mary was seen as a guide, mediator and advocate to God and a role model for devotion (Stöckigt et al., 2021).

The data from the interviews indicated health-, well-being-, resilience- and resource-strengthening effects, for example, confidence, relaxation, connectedness, prosocial behavior, strengthening of faith and promotion of peace. The prayer practice may help participants to better cope with everyday challenges, stressful situations, critical life events and illness.

Based on our qualitative preliminary study, we aim to use this follow-up cross-sectional study to identify the sociodemographic characteristics of those who pray the Rosary in Germany, their religion and spirituality (R/S), their prayer practice and how their religious/spiritual experiences and concepts relate to their subjectively perceived well-being. Furthermore, we aim to contribute to the rather superordinate research question of whether and how R/S and well-being are related. Based on a large body of empirical data, it can be assumed that R/S and well-being and health are slightly positively correlated. For example, Lefevor et al. (2021) recently compiled 20 meta-analyses that taken together showed a mean correlation of *r* = 0.15 between various R/S measures and health measures. In another meta-analysis, Hodapp and Zwingmann (2019) report a lower correlation (*r* = 0.03) between R/S and mental health with respect to studies from secularized German-speaking countries. We hypothesize that the correlation between R/S and well-being is higher in our sample of participants who pray the Rosary than in Hodapp and Zwingmann’s meta-analysis because it is presumably a highly religious and relatively culturally homogeneous sample. In particular, the question arises whether and how the experience of praying the Rosary statistically affects the (generally small positive) relationship between R/S and well-being: Does the positive experience of praying the Rosary have a moderating effect on the R/S-well-being connection? That is the connection more pronounced in those who have more intense positive experiences of prayer than in those who experience praying the Rosary less intensely?

## Methods

### Design

An online-based anonymous cross-sectional study was conducted. The online questionnaire was implemented using the SoSci Survey tool (Leiner, 2019) and made publicly available to participants on the server of the Charité–Universitätsmedizin Berlin.

### Ethics and Data Protection

The ethics board of Charité–Universitätsmedizin Berlin agreed to this study and the protocol on March 1st, 2022 (EA2/020/22). The study also received approval from the Charité data protection board.

### Participants

Inclusion criteria that were applied were age ≥ 18 years, prayer practice with the Rosary for at least 6 months, no existing dependence on the interviewing institute or their staff. The only exclusion criterion applied was lack of knowledge of the German language.

### Recruitment Strategy

A link to an online survey was distributed in Germany via mailing lists and online advertising. For this purpose, contact persons and multipliers in German dioceses, parishes, spiritual communities (e.g., monasteries), web portals (e.g., Missio, medjugorje.de, etc.) and Christian support organizations (e.g., Cusanuswerk) were contacted. In addition, we also recruited directly through church congregations. As an incentive, a 20 Euro voucher for books was offered to participants. For this purpose, those interested in the incentive had to provide a contact option and their consent for the use of their contact details.

### Rationale for Selecting the Measurement Instruments

The following criteria were taken into account when selecting the survey instruments:

1. The items of the R/S scales should be as close as possible to the formulations that were expressed by the participants in the qualitative preliminary study (Stöckigt et al., 2021). This was also intended to create a content-related fit between the instruments and the specific religious world view of the group of Rosary prayers. For example, talk of God is common in this Christian sample, which is why many items were selected that explicitly refers to God (which may be problematic in other, more diverse study populations).
2. The R/S scales were intended to reflect religiosity *and* spirituality. Therefore, when selecting the scales and questionnaire items, attention was paid to the nuances of meaning and conceptual differences. The definition of religion and spirituality by Jeserich et al. (2023) and the classification of R/S items and scales presented in this study served as orientation.
3. Religiosity/spirituality was to be recorded in a certain multidimensionality, taking into account those aspects of R/S that were mentioned particularly prominently in the qualitative preliminary study. These include the R/S aspects of meaning, trust, coping and experience. The selection of suitable items/scales was again based on the work of Jeserich et al. (2023).
4. The selected R/S scales should not touch on the aspect of well-being, as this would distort the correlation between R/S and well-being (for this problem, see e.g., Koenig, 2008).
5. To measure well-being, an instrument should be selected that is multidimensional, that is, covers physical, psychological and social aspects. However, it should not include the spiritual dimension of well-being, as this could lead to overlaps with the R/S scales.

### Measurement Instruments

The following questionnaires were used for the online survey:

#### Sociodemographic Data

Participants' sociodemographic data were collected using 11 items covering the following aspects: Year of birth, biological sex, highest educational qualification, employment status, marital status, nationality, religious affiliation, prevalence of recent critical life events and health status. The question regarding the participants’ health status was taken, with minor modification, from a health survey by the Robert Koch Institute (2017).

#### Prayer Practice

In order to learn more about the practice and the subjective experiences and concepts of Rosary prayers, we developed items on 8 topics ourselves: (1) we asked who the respondents learned the Rosary from (11 answer options, including an open answer field); (2) we recorded whether there was a trigger for the engagement with the Rosary (yes/no) and what life event it was (9 options, including an open answer field); (3) frequency of the prayer (5-point Likert scale from "rare and irregular" to "several times a day"); (4) type of Rosary (14 answer options, including an open answer field); (5) social context of prayer (5-point Likert scale from "group only" to "private only"); (6) concept of the Mother of God/Role of Mary (11 answer options, including an open answer field); (7) motivation for prayer (12 answer options, including an open answer field); (8) positive/negative experiences during prayer (9 statements on physical experiences, 6 on spiritual, 5 on emotional and 6 on cognitive; to answer on a 5-point Likert scale, ranging from "not at all true" to "completely true"). The answer options were based on the data we collected in our preliminary qualitative study (Stöckigt et al., 2021).

#### Positive Experiences During Rosary Practice

The statements which clearly described positive experiences during Rosary Prayer and which fit together due to psychometric characteristics were combined in a scale. This scale contains six statements relating to positive physical experiences, four statements relating to positive spiritual experiences, two statements relating to positive feelings and two statements relating to a positive state of mind during Rosary prayer practice. Agreement with the statements could be reported on a 5-point Likert scale (with response options ranging from "not at all true" to "completely true"), with a high mean score between 1 and 5 indicating a high expression of positive prayer experiences. Cronbach's α for the self-developed scale *Positive Experiences Rosary* is 0.88.

#### Level of Religiosity/Spirituality

The purpose of this single item was to find out how religious/spiritual the participants consider themselves to be. The response options on a 5-point Likert scale range from "not at all religious/spiritual" to "very religious/spiritual.” A distinction between the constructs “religion” and “spirituality” was not made at this point. The wording of the item is roughly based on question 11 of the “Religionsmonitor 2008,” a large representative survey conducted by the Bertelsmann Foundation in Germany (Huber, 2010).

#### Transpersonal Trust

The scale measures how high a person rates his or her own trust in something or someone transcendent (higher reality, higher being, God). The first three items from Belschner's (1998) 11-question single-factor scale *Transpersonales Vertrauen* (TPV) were specifically selected to focus on the issue of trust. For this reason, we did not use the psychometrically verified 4-item short scale of Hampel et al. (2020), which was constructed using other criteria. The four-point response scale of the original instrument was transformed into a 5-point Likert scale to make it compatible with the other measures of religion and spirituality. The range is thus between 1 and 5, with a high mean score indicating strong transpersonal trust. In a sample of German students, the TPV total scale achieved a Cronbach's α of 0.90 (Klein et al., 2012); in our study, the value for our 3-item short scale was 0.92.

#### Spiritual Meaning

The scale captures the extent to which respondents believe their spirituality helps give meaning and purpose to their lives. For this specific purpose, three items were selected from the *Multidimensional Measurement of Religiousness/Spirituality for Use in Health Research* (Fetzer Institute & National Institute on Aging Working Group 2003) that explicitly use the terms "spirituality" or "spiritual" (not “religiosity” or “God”) and that clearly focus on the function of meaning-making. These are items 7, 15 and 20 proposed by Pargament (2003) for the *Meaning-Long Form* subscale of the *Multidimensional Measurement of Religiousness/Spirituality*. The questions are answered on a 5-point Likert scale ranging from “strongly disagree” to “strongly agree.” A high total score means that spiritual meaning-making is an important factor in the respondent's life. In the absence of a German translation of the items, the three selected questions were translated into German by one of the authors (FJ) and reviewed by the author team. Our *Spiritual Meaning* scale achieved a Cronbach's α of 0.85 in the sample of Rosary practitioners.

#### Experience of God

The scale captures how often respondents subjectively feel they experience God's presence in their lives. The scale consists of three items taken from a subscale of Huber's *Religiositäts-Struktur-Test* (Huber, 2007), which was also used in combination with the *Religionsmonitor 2008* (Huber, 2010) and proved to be a reliable short scale (Huber, 2004). The questions are answered on a 5-point Likert scale with response options ranging from "never" to "very often." A high score means that the respondent experiences God frequently. Huber and Huber (2012) report in an article, which also includes an English translation of the three questions we selected (items 5, 10 and 15 of the CRS-15), that the internal consistency of the scales is sufficiently good (α values between 0.84 and 0.93). In the present study, Cronbach's α is 0.82.

#### Religiosity/Spirituality

This scale includes 9 items and is composed of the three previous scales: Transpersonal Trust (3 items), Spiritual Meaning (3 items) and Experience of God (3 items). It thus summarizes the three areas of R/S that seem to be relevant indicators of the religious/spiritual mindset of people who pray the Rosary, based on preliminary theoretical considerations and qualitative results (Stöckigt et al., 2021). The mean score of the scale ranges from 1 to 5, with a high score indicating higher religiosity/spirituality. Cronbach's α is 0.93.

#### Religious Problem-Solving Style

The scale is a self-construction based both on earlier theoretical constructs and on the considerations we made in our qualitative study of praying the Rosary. It includes five statements that respondents can agree with to varying degrees on a 5-point Likert scale (ranging from "strongly disagree" to "strongly agree"). Each statement describes a specific positive religious problem-solving style that correlates with a particular concept of God: collaborating (solving the problem together with God), deferring (leaving problem-solving to God), self-directing (trying to solve the problem without God's assistance), surrendering (actively choosing to follow the Divine Will) and balancing (balancing one's own actions with reliance on God). The first three styles were introduced by Pargament et al. (1988), Wong-McDonald and Gorsuch (2000) added the fourth style, and the fifth style emerged from our polar model (Stöckigt et al., 2021). Given that we asked about general religious problem-solving styles that might be mutually exclusive from the basic attitude and underlying image of God, it makes little sense to calculate a mean or total score for the items. Rather, we need to ask which of the five styles is the predominant one in the sample.

#### Well-being

*The General Habitual Well-Being Questionnaire* was developed by Wydra (2020) and is currently only available in German (*Fragebogen zum allgemeinen habituellen Wohlbefinden*, FAHW). The scale maps positive and negative aspects of well-being in the somatic, psychological and social domains. Despite this theoretical tripartite division, only the total value is calculated for the FAHW. The short version used here consists of 12 items to be answered on a 5-point Likert scale. Accordingly, the mean values range from 1–5 points, with items recoded so that high scores indicate high well-being. According to Wydra (2020, p. 55), the mean inter-item correlation is 0.35 and Cronbach's *α* is 0.86. Cronbach's *α* is 0.87 in the present study. Thus, the internal consistency can be rated as good.

#### Data Management and Procedures

Before the anonymous online survey began for a participant, a question ensured that the study participants agreed to the explained framework of the study and the use of their data. If this question was answered negatively, the online survey ended. The participants were informed about the nature and aim of the survey and about the data protection aspects of the survey. This was followed by consent or refusal to participate. Without the corresponding click and the consent given to the survey, the interested parties could not complete the questionnaire.

Only anonymous data were collected during the survey. After filling in the questionnaire, the data were transferred to the Charité server via an HTTPS connection (encryption) immediately after the Internet connection was established and was not stored on the computer/tablet/mobile phone. The entered data were to be downloaded regularly (2–3 times per week) from the Charité web server as an SPSS file and stored in a project folder of the Institute of Social Medicine, Epidemiology and Health Economics on a protected drive where access rights are highly restricted. The download was done via a password-protected HTTPS address, which could only be accessed from the Charité via a web browser. The security guidelines of the Quality Management Manual of the Institute of Social Medicine, Epidemiology and Health Economics were applied. The data will be stored securely for at least 10 years.

#### Statistics

We initially estimated that approximately 500 people who pray the Rosary could be reached through the available multipliers, it was estimated that from this group approximately 25% of Rosary praying people would fill out the online questionnaire, which would correspond to 125 participants. Data were exported from the database to SPSS and descriptively analyzed. Descriptive statistics were presented as frequency and percentage of qualitative variables, mean and standard deviation.

To also test whether the relationship between R/S and well-being is moderated by positive experiences while praying the Rosary, we proceeded with the following steps: first, we built two groups by dichotomizing (0/1) the scale *Positive Experiences Rosary* (PER). The group coded “0” comprises persons with only few or medium positive experiences (PER ≤ 3) and the group coded “1” is made up of persons with high expressions of positive prayer experiences (PER > 3). Second, for descriptive purposes only, we calculated the correlation between R/S and *Well-being* within each of the two groups separately. Third, a 2-step hierarchical linear regression analysis was conducted with *Well-being* as the criterion variable. After z-standardizing, the metric predictor R/S, R/S and the dichotomized PER (0/1) were entered in Step 1, and the interaction of these two variables was entered in Step 2. Due to the (0/1)-coding PER can affect *Well-being* only if persons report high expressions of positive prayer experiences (Urban & Mayerl, 2018). Thus, if the interaction term adds significant predictive variance to the regression model, it can be concluded that positive experiences during the Rosary prayer act as a moderator variable.

## Results

### Recruitment

The online questionnaire was filled out by participants from June 10th until September 15th, 2022. In total, 206 persons gave their consent to participate, out of those 164 (79.6%) completed the whole questionnaire and their data could be analyzed.

### Sociodemographic Data

The sociodemographic data of the respondents is presented in Table 1. The majority of the participants were between 40 and 59 years (50.6%) and 60–79 years (32.5%) of age, and 61% of the sample consisted of women. In total, 92.4% of the participants belonged to the Roman Catholic Church. A majority of participants reported to be very religious/spiritual (35.8%) or quite religious/spiritual (46.7%), only 13.9% reported to be medium religious and 3.7% little or not religious.

**Table 1:** Sociodemographic data

Item/characteristics Total: *n* = 164	*n*	Frequency (%)
*Gender*		
Female	100	61
Male	64	39
Diverse	0	0
*Age (years) (n = 160)*		
> 80	4	2.5
60–79	52	32.5
40–59	81	50.6
20–39	23	14.4
< 20	0	0
*Highest educational qualification (n = 163)*		
University degree	82	50.3
High school or equivalent	37	22.7
≤ 10 years of school	40	24.5
others	4	2,5
*Profession (n = 163)*		
Employed	64	39.3
Self-employed	31	19
Civil servant	16	9.8
Retired	29	17.8
Student	2	1.2
Unemployed	2	1.2
Housewife/husband	13	8
Other	6	3.7
*Marital status (n = 163)*		
Married	86	52.8
Other partnership	8	4.9
Single	43	26.4
Divorced/living apart	17	10.4
*Nationality (n = 164)*		
German	151	92
*Living region (n = 163)*		
Urban	101	62
Rural	62	38
*Religion (n = 157)*		
Roman catholic	145	92.4
Protestant (several)	10	6.3
Other	2	1.3
*Significant life events experienced in the last 4 months (n = 162)*		
Positive life event	31	19.1
Negative life event	27	16.7
Negative and positive life event	15	9.3
None	89	55
*Chronic disease (more than 6 months) (n = 162)*		
Yes	54	32.9
No	108	65.9

Most participants reported a high level of education with 50.3% stating university degree and 22.7% high school as highest reached level, only 24.5% reported a maximum of 10 years of school. Professionally, most reported to be working, with 39.3% being employed and 19% self-employed, only 17.8% were retired. Half of the participants were married (52.8%), 92% reported German nationality, and 62% lived in an urban region. Regarding disease and life events, 32.9% reported having a chronic disease and 19.1% had experienced a negative life event within the last 4 months (especially COVID 19 disease or death of family or friends), 16.7% described a positive life event (e.g., being together again with friends after COVID 19, religious experience, birth) and 9.3% positive and negative life events combined.

### Prayer Practice

Data about the practice of the Rosary prayer are presented in Table 2. Most participants learned about the Rosary prayer in their church community (45.7%), from their mother (36%), grandmother (26.8%) or on a pilgrimage (26.2%). One third reported that a specific trigger caused them to start praying (34.8%); most often those were stressful life events, specific persons or spiritual experiences. Almost half of the participants (42.7%) pray the Rosary regularly (daily or several times a week), while 57.3% practice the Rosary prayer irregularly to rarely. Most often the classic rosary (59 beads) is used for prayer (70.7%) followed by the finger rosary (22.6%) and others. Approximately, one third of the respondents reported to pray mainly, mostly or at least half of the time in a prayer group, the COVID pandemic did not change that.

**Table 2:** Prayer practice

Item	*n*	%
*How did you get to know the Rosary prayer? (n = 147)*		
Church community	75	45.7
Mother	59	36
Grandmother	44	26.8
Pilgrimage	43	26.2
Friends and acquaintances	35	21.3
Father	34	20.7
Other family members	24	14.6
Literature	14	8.5
Internet	12	7.3
Grandfather	10	6.1
Others	16	9.8
*Was there a trigger for getting involved with the Rosary prayer? (n = 145)*		
Yes, there was a trigger	57	34.8
Stressful life event	24	14.6
Specific person	17	10.4
Spiritual experience	16	9.8
Physical illness	9	5.5
Healing experience	7	4.3
Mental illness	5	3.0
Positive life event	5	3.0
Spiritual crisis	4	2.4
Other	12	7.3
*Frequency of praying the Rosary (n = 145)*		
Daily	43	26.2
Several times a week	27	16.5
Infrequent and irregular	23	14.0
Only on certain occasions	19	11.6
Once a week	17	10.4
Several times a day	9	5.5
Once a month	7	4.3
*Which rosary do you pray? (n = 140)*		
Classic rosary (59 beads)	116	70.7
Finger rosary	37	22.6
Mercy rosary	38	23.2
Wound rosary	8	4.9
*Prayer always, mostly or often in a group (n = 144)*		
At present	51	35.7
Before COVID 19 pandemic	50	34.7

### Positive/Negative Experiences During Prayer

Table 3 shows the descriptive statistics of the prayer experience, which is how often participants have certain emotional, cognitive, physical and spiritual experiences while praying the Rosary. The physical experiences during prayer are essentially associated with rhythmic breathing (rather true, or completely true 50.6%), slowing of breathing (42.7%), relaxation (53%) and perception of the prayer beads (63.4%). The spiritual experiences include the feeling of entering a sacred space (34.1%) and feeling of divine presence (36%). The most important emotional experiences are coming to rest inwardly (71.9%) and feeling happier (42.1%), cognitive experiences are forgetting worries (41.5%). The 14 items on positive prayer experiences with the highest item-total correlations were combined to form the *Positive Experiences Rosary* scale. The mean score of this scale is 2.98 ± 0.7 (range 1–5) (Table 6).

**Table 3:** Religiosity/spirituality measures

	Not at all true/never (%)	Rather does not apply/seldom (%)	Sometimes applies/sometimes (%)	Rather true/frequently (%)	Completely true/very frequently (%)	Not answered or missing (%)
*Rosary prayer experiences*						
Physical						
I breath rhythmically*	5.5	7.9	15.9	31.7	18.9	20.1
My muscles relax*	5.5	7.3	15.9	33.5	19.5	18.3
I no longer feel my body*	31.1	28.0	11.0	6.1	1.8	22.0
My breath slows down*	10.4	11.0	15.2	29.3	13.4	20.7
I am consciously aware of my breath*	14.6	20.7	16.5	15.2	9.8	23.2
I feel the beads of the rosary in my hand*	5.5	3.7	9.1	27.4	36.0	18.3
I have the feeling my heart beats faster	47.0	25.0	4.9	0.0	2.0	22.0
My physical pain is increasing	59.8	12.8	3.0	0.0	1.8	22.6
Spiritual						
It is as if I am entering a sacred space*	14.6	12.2	18.9	26.2	7.9	20.1
I feel the divine presence*	10.4	6.7	28.0	22.6	13.4	18.9
I have religious visions or auditions*	53.0	12.2	7.9	2.4	1.2	23.2
I experience a trance-like state*	48.8	18.3	5.5	4.3	0.6	22.6
I feel threatened by evil forces	65.2	6.7	3.7	1.2	0.6	22.6
Emotional						
I come to rest inwardly*	3.7	0.0	7.9	39.0	32.9	16.5
My feelings go crazy	59.8	14.0	3.0	0.6	1.2	21.3
I feel fear and guilt	62.2	10.4	3.0	1.2	1.2	22.0
I am happy*	4.3	3.0	29.9	32.3	9.8	20.7
Cognitive						
I become completely empty	26.8	22.0	14.6	11.0	3.7	22.0
I am in a state of loose attention*	16.5	14.6	19.5	23.2	6.1	20.1
My thoughts are racing	48.2	18.3	5.5	5.5	0.6	22.0
I am thinking about my life	11.0	9.1	24.4	25.0	9.8	20.7
I forget my worries*	6.7	9.8	23.8	35.4	6.1	18.3
*Religiosity/Spirituality (combined scale with 9 items)*						
Transpersonal trust						
I feel connected to a higher being**/**God. I can trust in this even in difficult times	3.7	2.4	N/A	25.6	50.6	17.7
I try to entrust myself to the hand of God**/**a higher being**/**a higher reality	3.0	2.4	N/A	25.6	50.6	18.3
We humans cannot determine everything. There is a higher reality**/**a higher being**/**God, to whom I can entrust myself	3.0	2.4	N/A	17.1	60.4	17.1
Spiritual meaning						
My spiritual beliefs give my life a sense of significance and purpose	3.0	0.0	1.8	29.3	49.4	16.5
Looking at the most troubling or confusing events from a spiritual perspective adds meaning to my life	5.5	3.0	14.0	29.3	30.5	17.7
My spirituality helps define the goals I set for myself	3.7	0.6	9.1	40.9	28.7	17.1
Experience of God						
How often do you experience situations in which you have the feeling that God or something divine intervenes in your life?	4.3	7.3	31.7	28.7	11.0	17.1
How often do you experience situations in which you have the feeling that God or something divine wants to communicate or to reveal something to you?	3.7	11.6	32.2	29.3	6.7	16.5
How often do you experience situations in which you have the feeling that God or something divine is present?	3.0	9.1	19.5	31.1	20.1	17.1
*Religious Problem-Solving Style*						
God**/**a higher power and I work hand in hand to solve my problems	4.9	9.1	17.7	29.9	18.9	19.5
I trustfully leave the solution of my problems to God**/**a higher power	6.1	6.7	28.7	23.2	17.1	18.3
I try to solve my problems without the help of God or a higher power	29.3	31.1	14.0	3.7	3.7	18.3
Even if there is no solution to my problems, I continue to do what I feel is divine will or plan	4.9	7.3	11.0	39.6	17.1	20.1

The symbol * indicates that the item is part of the Positive Experience Rosary (PER) scale

### Role of Mary

Data on the role of Mary are presented in Table 4. Most participants venerated Mary as a mother, as the woman who gave birth to the Son of God (completely true: 64%). Participants considered Mary as a mediator between God and men (completely true: 50%), as an advocate for “us sinners” (completely true: 44.5%) and that Mary shows the way to Jesus Christ (completely true: 51.8%). More ambivalent answers were given to the questions of whether Mary is a close friend or whether she is approachable and experienceable (for more data see Table 4).

**Table 4:** Role of Mary

Role of Mary	Not at all true (%)	Rather does not apply (%)	Sometimes applies (%)	Rather true (%)	Completely true (%)	Not answered and missing (%)
I venerate Mary as Mother, as the woman who gave birth to the Son of God	4.9	0.6	1.2	13.4	64.0	15.9
Mary is an advocate for us sinners	8.5	5.5	4.3	17.1	44.5	20.1
Mary was a brave and strong woman who endured much pain and suffering	3.7	1.2	3.7	14.6	57.9	18.9
Mary is a mediator between God and people	9.8	2.4	6.7	14.0	50.0	17.1
As a virgin, Mary is a model of virtue	12.2	9.8	6.1	14.6	36.6	20.7
Mary shows us the way and leads us to Jesus Christ	6.1	2.4	7.9	12.8	51.8	18.9
Mary embodies values of purity, humility and piety	5.5	2.4	13.4	15.2	42.7	20.7
Mary is like a close friend to me	7.9	14.0	17.7	17.7	23.8	18.9
Mary is approachable and experienceable	4.9	4.3	11.6	25.0	35.4	18.9
Mary does not play a special role in my faith	52.4	14.0	4.9	2.4	6.1	20.1

### Transpersonal Trust

With a mean of 4.40 ± 0.91 (range 1–5) (Table 6), the respondents showed high values in the area of transpersonal trust: more than half feel connected to God (completely true: 50.6%), entrust themselves to the hands of God (completely true: 50.6%) and believe that there is a higher being/God to whom they can entrust themselves (completely true: 60.4%).

### Spiritual Meaning

Regarding spiritual meaning, the sample of Rosary prayers showed a very high mean score of 4.16 ± 0.86 (range 1–5) (Table 6). Seventy-nine percent of the sample agree or strongly agree that their spiritual beliefs give their lives meaning and purpose. A very high percentage of the participants (70%) agree or strongly agree that their spirituality helps to define goals being set for themselves, 60% agree or strongly agree with “looking at the most troubling or confusing events from a spiritual perspective adds meaning to my life.”

### Experience of God

The mean score for the experience of God is 3.46 ± 0.86 (range 1–5) (Table 6). Situations in which they have the feeling that God wants to tell them something are often and very often experienced by 36% of respondents, occasionally by 32.3%.

Situations in which they have the feeling that God is intervening in their lives are often or very often mentioned by 40%, occasionally by 31.7%. The feeling that God is present is felt often or very often by 51%, and occasionally by 19.5% of respondents.

### Level of Religiosity/Spirituality

The Rosary prayers describe a high level of religiosity/spirituality with a mean score of 4.0 ± 0.78 (range 1–5) (Table 6): 29.9% of respondents describe themselves as very religious/spiritual, 39.0% as fairly religious and 11.6% as moderately religious.

#### Religious Problem-Solving Style

For many participants, trust in God was an important strategy for coping with difficult situations (Table 3). It is evident that the participants do not try to solve their problems on their own, but prefer two different religious coping strategies: either they hand over the solution to the problem entirely to God (17.1% completely true, 23.2% rather true) or they assume an interplay between divine and human actions (18.9% completely true, 29.9% rather true). The majority continue to practice prayer with confidence, even if no solution to their problems emerges.

#### Well-Being

Table 5 shows the descriptive data on *The General Habitual Well-Being Questionnaire*. The majority of the Rosary prayers describe a high state of well-being, the mean score for well-being was 3.88 ± 0.68 (range 1–5) (Table 6). More than half (60.1%) describe themselves as physically healthy (“completely” or “something like this”), 56.6% feel balanced and 57.3% feel in tune with the state of their body. Only a minority describes problematic aspects like depressed mood (10.4%), constant pain (12.2%) or disappointment in fellow human beings (7.3%).

**Table 5:** Well-being

	Yes, exactly like this (%)	Something like this (%)	I do not know (%)	Not like this (%)	Certainly not like this (%)	Not answered and missing (%)
I am very balanced	11.6	45.1	14.6	7.3	3.0	18.3
I can approach others without problems	34.1	34.1	5.5	5.5	2.4	18.3
When I move I can feel my illness	6.7	6.7	5.5	22.0	40.9	18.3
I feel abandoned	1.8	5.5	7.9	18.9	47.6	18.3
I am constantly in pain	3.7	8.5	3.0	14.0	51.8	18.9
I feel in tune with the state of my body	20.1	37.2	10.4	11.0	2.4	18.9
I have lots of friends	15.9	32.9	15.9	15.9	1.2	18.3
I've got everything under control	1.8	31.1	19.5	19.5	9.8	18.3
My mood is depressed	3.7	6.7	5.5	41.5	23.8	18.9
I am disappointed in my fellow human beings	0.6	6.7	16.5	37.8	19.5	18.9
I feel physically healthy	22.9	37.2	6.7	9.1	5.5	18.9
I can no longer stand the inner tension	0.6	5.5	7.9	17.1	50.0	18.9

**Table 6:** Means, SDs, and intercorrelations of religious/spiritual scales, well-being and *Positive Experiences Rosary*, 123 ≤ *N* ≤ 136

		Theoretical range	Mean	SD	1	2	3	4	5
1	Transpersonal trust	1–5	4.40	0.91	–				
2	Spiritual meaning	1–5	4.16	0.86	0.78**	–			
3	Experience of god	1–5	3.46	0.86	0.64**	0.65**	–		
4	Religiosity/spirituality	1–5	4.00	0.78	0.91**	0.91**	0.85**	–	
5	Well-being	1–5	3.88	0.68	0.19*	0.20*	0.10	0.19*	–
6	Positive experiences rosary (PER)	1–5	2.98	0.70	0.51**	0.55**	0.52**	0.59**	0.04

^*^ *p* < .05, ** *p* < .01

#### Religion/Spirituality and Well-Being

All three of the Religiosity/Spirituality (R/S) scales Transpersonal Trust, Spiritual Meaning and Experience of God as well as the combined scale Religiosity/Spirituality are highly intercorrelated (*r* between 0.64 and 0.91; see Table 6) and show similar small-size associations (*r* between 0.10 and 0.20) with well-being (Cohen, 1988). Hence, it is reasonable to conduct the moderator analysis with the combined scale R/S only. The scale *Positive Experiences Rosary* is not significantly connected with well-being (*r* = 0.04). Calculating the correlation between R/S and well-being for persons with low (*r* = 0.08, *p* = 0.514, *n* = 63) vs. high expressions (*r* = 0.37, *p* = 0.003, *n* = 60) of positive prayer experiences separately reveals a pronounced difference which strongly suggests that the connection between R/S and well-being was stronger among those with more positive prayer experiences. This is confirmed by the hierarchical regression analysis with well-being as criterion variable. The results are shown in Table 7. In Model 2, the Religiosity/Spirituality × Prayer Experience interaction is not only a significant predictor of well-being (β = 0.255, *p* = 0.020) but also the only significant predictor. That is, only in those who report high levels of positive prayer experience, is increased R/S associated with higher well-being. Model 2 only accounts for 7.6% of the variance in well-being (adjusted R^2^ = 0.053).

**Table 7:** Hierarchical regression summary predicting well-being (*N* = 123)

	Model 1		Model 2	
	*β*	*p*	*β*	*p*
Step 1 (enter)				
Religiosity/spirituality R/S	0.183	0.060	0.071	0.505
Dichotomized positive experience rosary PER (0/1)	–0.009	0.930	–0.070	0.475
Step 2 (enter)				
R/S × PER (0/1)			0.255	0.020
R^2^ change	0.033	0.138	0.044	0.020
R^2^	0.033	0.138	0.076	0.024
Adjusted R^2^	0.016		0.053	

## Discussion

To our knowledge, this is the first cross-sectional study investigating the topic of Rosary prayer and well-being. Our sample consisted mainly of well-educated German Roman Catholics (92%), with 61% being women. Most participants learned the Rosary prayer from their mother or grandmother, but also in their church communities and on pilgrimages. Approximately one third (35%) of the participants described a specific trigger which led to the practice of Rosary prayer in the past, in 15% this was a stressful life event. Most respondents pray the Rosary daily or several times per week, and R/S plays a central role in their lives. They describe very high levels of R/S, a high trust in God, high levels of spiritual meaning and overall, a high level of well-being. However, it also shows that trusting prayer to God is not the only problem-solving strategy for this specific population. Many participants agree that letting go (trusting God) and doing (own action) must go hand in hand in order to master difficult situations in life (cf. the dual coping model in Stöckigt et al., 2021).

As expected, the correlations between R/S variables and well-being in our study are significantly higher (*r* between 0.10 and 0.20) than for the German-speaking area (*r* = 0.03) according to the meta-analysis by Hodapp and Zwingmann (2019). This indicates that we are dealing with an atypical sample for secular Germany, namely a highly religious/spiritual one. In fact, the mean values of all religious/spiritual scales are in the upper range (for the combined scale Religiosity/Spirituality *M* = 4 on a five-point scale) (compare Table 6).

With regard to the physical, spiritual, emotional and cognitive experiences gained during praying the Rosary, the mean value is only in the middle range (*M* = 2.98 on a five-point scale). In fact, two approximately equal groups can be formed: individuals without (*n* = 63) and individuals with (*n* = 60) markedly positive experiences during Rosary prayer. For the group with markedly positive experiences, we find a significantly higher correlation (*r* = 0.37) between R/S and well-being than for the group with few positive experiences (*r* = 0.08). A hierarchical regression analysis shows that positive experiences during Rosary prayer do indeed moderate the relationship between R/S and well-being. In this analysis, the moderating interaction term is the only significant predictor of well-being. This means that only in persons who have positive experiences during the Rosary prayer is a significant correlation between R/S and well-being shown. According to Cohen (1988), this lies in the medium range (*r* = 0.37).

Overall, our data indicate that positive experiences during Rosary prayer are hardly associated with well-being (*r* = 0.04). However, they apparently show that a basic religious/spiritual attitude is associated with well-being. When people have positive (physical, emotional, cognitive and spiritual) experiences during Rosary prayer, this increases the likelihood that factors such as transpersonal trust, God relationship or religious/spiritual meaning-making relate positively to a person’s overall well-being. It can be assumed that subjectively perceived positive prayer experiences act as an amplifier or emotional affirmation of the “rightness” or “effectiveness” of one’s faith and that this very amplification has a strengthening effect on the relationship between R/S and well-being.

In our previous qualitative study, we found that reported experiences during Rosary prayer and perceived effects, such as relaxation, calmness, feeling of peace and trust, equanimity and a connection to God may overlap and foster well-being. Additionally, the subjective explanations of the experiences during prayer have an impact on the perceived effects and well-being (Stöckigt et al., 2021). Kohls and colleagues have found that the link between R/S and well-being depends not only on whether or not someone practices spiritually/religiously, but also on what experience one has during religious/spiritual practice (Kohls et al., 2009). In another study, they have determined that negative spiritual experiences are a significant predictor of distress and that people who have positive spiritual experiences during their practice are less stressed and more mindful (Walach et al., 2009). Thus, it appears that positive/negative experiences during spiritual/religious practice are important moderators of the R/S-well-being connection. Studies with similar questions but different variables enrich the overall picture. Wnuk (2018), among others, found that frequency of prayer has a moderating effect on the positive association of God relationship and job satisfaction. Lazar (2015) observed that prayer duration has a moderating effect on the correlation of prayer and well-being among Jewish women when distinguishing whether or not subjects believe in their prayers: "For women with a high belief in prayer, a positive prayer-well-being relation emerged when prayer duration was long, and a negative prayer-well-being relation emerged when prayer duration was short. In contrast, for women with a low belief in prayer, the opposite pattern emerged; lengthy prayer was negatively related to well-being, whereas short prayer was positively related to well-being." Poloma and Pendleton (1991) denoted that the prayer experience has a strong impact on quality of life and well-being. Overall, more research is needed to determine whether and what (moderating) effect variables such as "prayer experience," "prayer frequency," "prayer duration" and "belief in prayer" have on the relationship between R/S and well-being.

With regard to practice, our findings might suggest that religious/spiritual practitioners and people offering spiritual/religious activities (such as mindfulness training, retreats, communal prayer sessions, meditation, etc.) to strengthen people's well-being should be sensitized to what positive and/or negative experiences are reported by practitioners. In line with this, and from a health psychology perspective, it is not only the spiritual/religious practice in and of itself that is crucial, but the way in which it is subjectively experienced.

### Strengths

A methodological strength of this survey is the questionnaire that was strictly developed and grounded on hypotheses that were generated and developed in our former qualitative interview study.

### Limitations

A limitation of the study is a comparably low number of included participants (*n* = 164). The online recruitment process included a variety of Catholic organizations running email newsletters or webpages, but may also have excluded older people without Internet access or affinity. Another limitation of our study might be a selection bias due to our digital recruitment strategy—we have not reached Rosary prayers without digital communication tools and the recruitment in local Catholic communities and prayer circles was much smaller than online. We may also have missed more non-Catholic Rosary practitioners which might exist in smaller numbers. Unfortunately, in our online questionnaire, we did not assess whether a participant was a priest, nun, religious brother or a lay person, which may have biased the results as well. However, approximately half of the participants were married. A daily prayer practice of 43% could be overestimated in case a higher percentage of participants were priests or nuns.

In addition, approximately 15–25% of the respondents did not completely answer certain questions or stopped answering certain questionnaires online. The most likely explanation—apart from technical problems with the Internet connection—is that the questions sometimes asked about very personal aspects of religiosity and spirituality and could lead to mistrust of the scientists conducting the survey, who were not personally known. Also, it should be emphasized that the hierarchical regression model only explains 7.6% of the variance in the well-being criterion. Thus, there remains considerable scope for other, presumably more important determinants of well-being. A further limitation of the current study is its cross-sectional design which does not allow for any causal explanations to be made. Therefore, conclusions based on causality cannot be drawn and are speculative.

However, the results of our analysis support and strengthen many of our hypotheses derived from our qualitative research. Especially the importance of women and family in teaching and practicing the prayer was also shown in our qualitative study. Also, the fact that the Rosary prayer is often started in times of stress and significant life events hints toward the prayers’ function as a spiritual resource and religious coping strategy.

## Conclusion

Positive prayer experiences may act as an amplifier or emotional affirmation of the "rightness" or "effectiveness" of one's faith. This amplification may have a strengthening effect on the relationship between R/S and well-being. The interaction of R/S and well-being in Rosary praying and other meditative techniques should be a major topic of future research.

## Data Availability

The datasets generated and analyzed in the current study are available from the corresponding author on reasonable request.
